# Advances in pathogenesis and nanoparticles (NPs)-mediated treatment of psoriasis

**DOI:** 10.3389/fimmu.2022.1089262

**Published:** 2022-12-22

**Authors:** Qian Shen, Rong Liu, Shiyu Tan, Xiaoding Xu, Junyue Fang, Rong Li

**Affiliations:** ^1^ Department of Pharmacy & Pharmacology and the Second Affiliated Hospital, Hengyang Medical School, University of South China, Hengyang, China; ^2^ Guangdong Provincial Key Laboratory of Malignant Tumor Epigenetics and Gene Regulation, Medical Research Center, Sun Yat-Sen Memorial Hospital, Sun Yat-Sen University, Guangzhou, China; ^3^ Cellular and Molecular Diagnostics Center, Sun Yat-Sen University, Guangzhou, Guangdong, China

**Keywords:** psoriasis, autoimmune disease, pathogenesis, nanoparticles, psoriasis treatment

## Abstract

Psoriasis is a chronic papulosquamous skin disease with an autoimmune pathogenic traits and strong genetic predisposition. In the past few decades, with the rapid development of molecular biology and cell biology, the inherent pathogenesis of psoriasis has been gradually elucidated, in which cytokine inflammatory loops, cell signaling pathways, and epigenetic factors such as miRNAs have been demonstrated to play important roles in regulating the development and progression of psoriasis. More importantly, understanding the pathogenesis of psoriasis has promoted the development of effective treatment for psoriasis. In this review, we systemically summarized the molecular mechanisms regulating the development and progression psoriasis, introduced various therapeutics used for clinical psoriasis therapy, and highlighted the recent advances in nanoparticles (NPs)-mediated drug delivery for psoriasis treatment.

## Introduction

Psoriasis is an immune-mediated and chronic-hereditary inflammatory skin disease, which affects around 2% of the global population and imposes a heavy psychological and physical burden on patients (1–3). The pathogenesis of psoriasis is multifactorial and the potential pathological mechanism involves complicated interactions between the adaptive and innate immune systems. Interactions between various types of immune cells and keratinocytes can trigger the secretion of cytokines such as interferon-γ (IFN-γ), tumor necrosis factor-alpha (TNF-α), and some cytokines of interlukin family (IL-1β, IL-6, IL-23, IL-23, *etc.*) (4–7). Multiple signaling pathways (*e.g.*, mTOR, JAK/STAT, and MAPK) are also involved in the pathogenesis and progression of psoriasis. In addition, emerging evidences have revealed that epigenetics especially microRNAs (miRNAs)-mediated target gene expression plays an important role in the development and progression of psoriasis.

At present, topical therapy and systemic therapy are first-line therapeutic modalities for the treatment of psoriasis. Topical therapy is usually used for the treatment of early psoriasis *via* the administration of vitamin D3 or glucocorticoids complemented with phototherapy. For the patients with advanced psoriasis, systemic therapy is the main therapeutic modality, which usually involves the use of methotrexate (MTX), cyclosporine A, or biotherapeutics targeting cytokines (*e.g.*, infliximab, adalimumab, etanercept, and ustekinumab) (8–10). In the past decade, although these therapeutics have made a great achievement in psoriasis treatment, some key issues are still unsolved and frequently encountered in clinic, especially their toxic and side effects, leading to suboptimal therapeutic outcomes. Clinical observations have shown that most of psoriasis patients received systemic therapy have upper respiratory tract infection, urinary tract infection or herpes simplex infections (11). Therefore, design and development of new effective therapeutic strategy with low toxicity could facilitate to improve the therapeutic outcomes of psoriasis patients.

Over the past few decades, nanoparticles (NPs)-mediated drug delivery has been widely used for the treatment of various diseases, showing the advantages of promoting drug water solubility, enhancing drug stability and bioavailability, reducing toxic and side effects, and improving therapeutic outcomes (12). Inspired by these unique advantages, the application of NPs for the delivery of anti-psoriasis drugs has opened a new era in the treatment of psoriasis. To date, various types of NPs have been developed for drug delivery and psoriasis therapy, which could not only improve the therapeutic effect of anti-psoriasis drugs, but also significantly weaken their toxic and side effects (13). In this review, we systematically summarized the pathogenesis of psoriasis, introduced the therapeutic modalities for psoriasis, and reviewed the recent advances in NPs-mediated drug delivery for the treatment of psoriasis.

## Pathogenesis of psoriasis

### Imbalance of cytokine profiles in psoriasis

**Figure 1:**
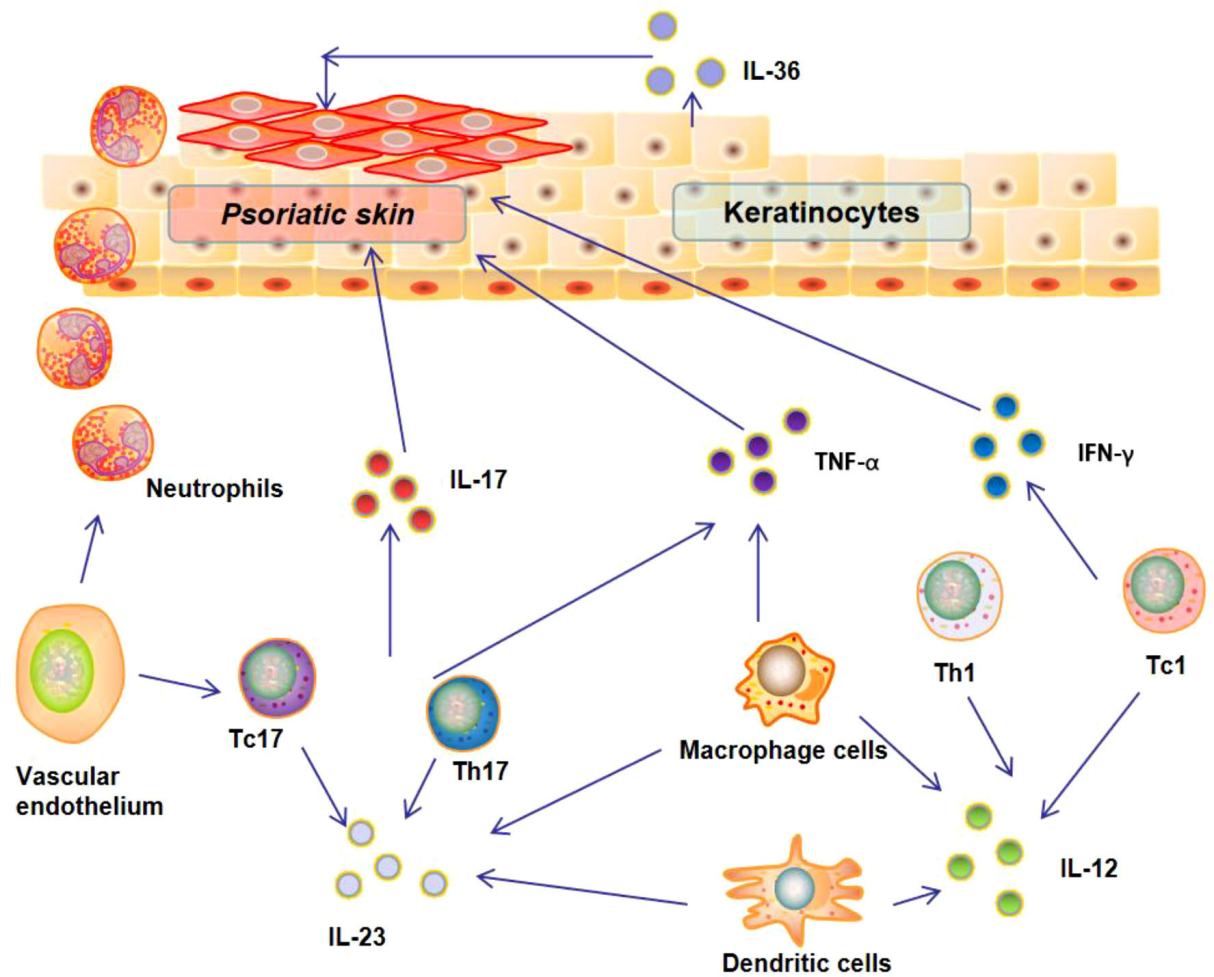
Inflammatory factor loop and immune dysfunction in psoriasis.

Over the past two decades, inflammatory circuit triggered by various cytokines (*e.g.*, IFN-γ, TNF-α, and IL-1β) have been found to play an important role in regulating the development and progression of psoriasis (**Figure 1**). Numerous researches have demonstrated that the interaction between various types of immune cells and keratinocytes could drive epidermal hyperproliferation and promote the production of cytokines, growth factors, and antimicrobial proteins (14). These factors collaborate and generate a self-sustaining cytokine inflammatory circuit to promote the progression of psoriasis (15).

INF-γ is an important effective immunomodulatory cytokine that mainly derived from T cells (16, 17). Numerous studies have revealed the elevated level of INF-γ in psoriasis patients (18, 19). This elevated IFN-γ could trigger macrophages to release high levels of other inflammatory cytokines and pro-inflammatory mediators, including chemokine (C-X-C motif) ligand 9 and 10 (CXCL9, CXCL10), which could in turn recruit T helper type 1 (Th1) and type 1 T cytotoxic (Tc1) cells to the psoriatic tissues and promote the progression of psoriasis (20). TNF-α is another cytokine up-regulated in psoriasis patients. In fact, TNF-α is a cytokine with pleiotropic effects on multiple cell types and involved in the pathogenesis of a variety of human autoimmune diseases (21, 22). It has been reported that keratinocytes in psoriasis patients are under stress and can secrete TNF-α and IL-6 to activate dendritic cells (DCs) to promote the progression of psoriasis. Due to the important role of TNF-α in the pathogenesis of psoriasis, TNF-α blockage has been used for clinical treatment of psoriasis patients. However, long-term TNF-α blockage could also induce side effects, especially increasing the risk of pediatric patient with psoriasis (23).

Besides INF-γ and TNF-α, some key cytokines of interleukin family have been also identified as the key factors in the pathogenesis of psoriasis (24). The representative ones are IL-1β and IL-6, which have been found to be up-regulated in psoriatic skin lesions (25, 26). The elevated level of IL-6 in psoriatic lesions could not only promote the proliferation of keratinocytes and the differentiation of IL-17-secreting cells, but also inhibit the differentiation of regulatory T Cells (Tregs) cells (24). Currently, targeting these two cytokines has been one of the strategies to treat various skin autoimmune diseases including psoriasis (27). IL-23, an important pro-inflammatory cytokine responsible for the differentiation and proliferation of T helper 17 (Th17) cells, has been also reported to involve the pathogenesis of psoriasis. IL-23 could trigger Th17 cells to secrete IL-17 and IL-21, which could enhance neutrophil infiltration into the psoriatic lesions and promote psoriasis progression (28). In addition, IL-36 has been also found to play a crucial role in regulating the progression of psoriasis (29, 30). A recent study revealed that the interaction between IL-36 and its receptor on keratinocytes could induce the secretion of IL-17and IL-21 to enhance neutrophil infiltration into the psoriatic lesions (31).

The role of other cytokines in psoriasis is often controversial, including IL-4, IL-10, and IL-13. For example, some studies found that IL-4 secreted by Th2 polarized T cells was increased in psoriasis patients (32, 33), while other scholars indicated that this cytokine was decreased psoriasis patients (34). Although the cytokines such as IL-4, IL-10, and IL-13 play a relevant role in the pathophysiology of psoriasis, the corresponding inhibitors in clinical trials have not yet achieved the expected efficacy. Anyway, it is undoubted that the balance of cytokine profile and cytokine signaling pathways play a central role in the pathogenesis of psoriasis. Multiple types of cytokines interact, amplify and activate to form a self-sustaining cytokine inflammatory loop (**Figure 1**). A deep understanding of this inflammatory loop is the basis for developing effective therapeutics for treatment.

## Main signaling pathways involved in psoriasis

### PI3K/AKT/mTOR signaling pathway

**Figure 2:**
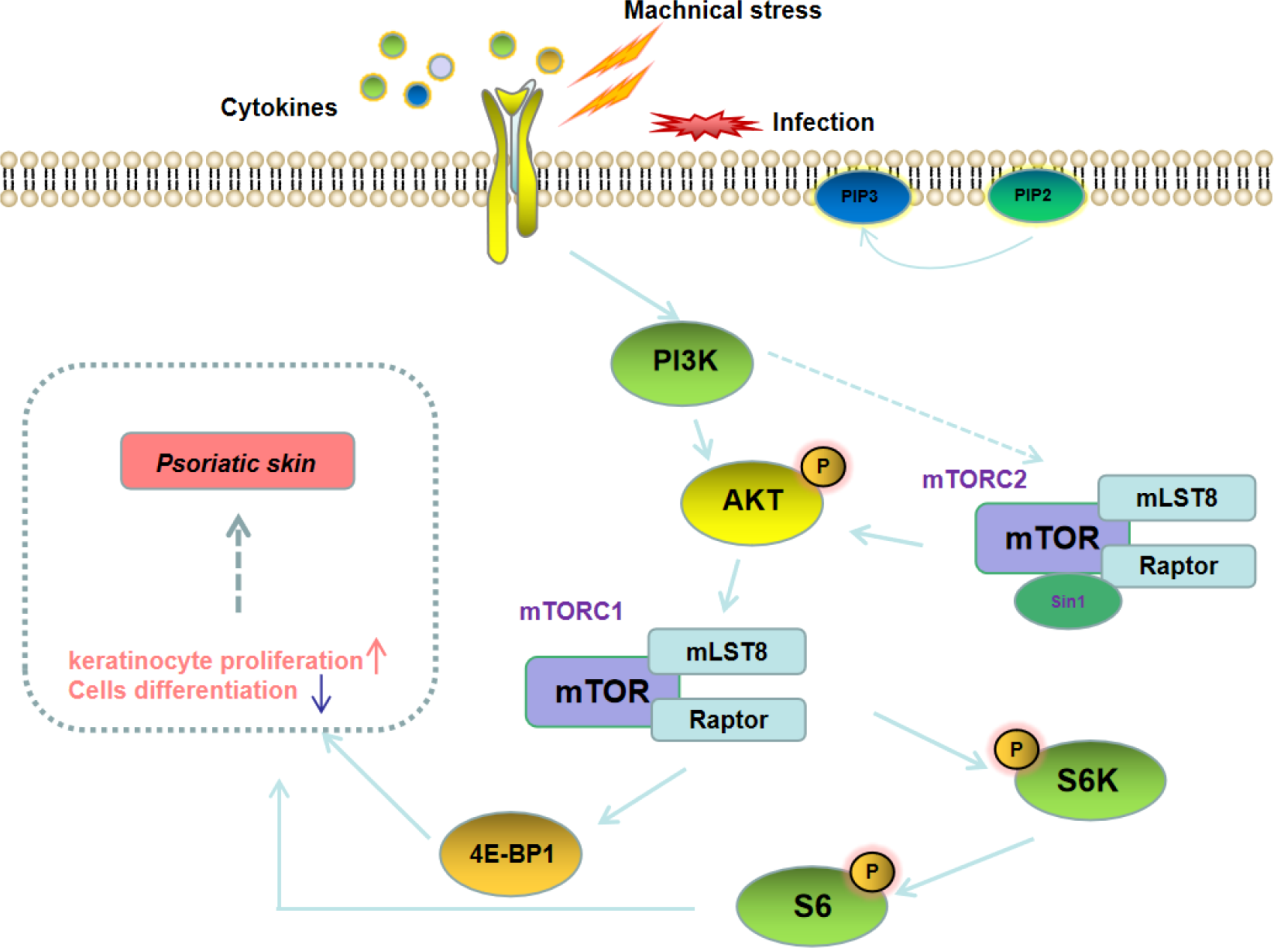
The PI3K/AKT/mTOR signaling cascade.

Mammalian target of rapamycin (mTOR) is an important signaling hub that coordinates cellular and tissue responses. The upstream pathways of mTOR include PI3K-AKT and LKB1/CD73-AMPK. The PI3K/AKT/mTOR signaling pathway is widely involved in the proliferation of various types of cells, and also plays an important role in regulating development and progression of autoimmune diseases including psoriasis (**Figure 2**) (35, 36). A study on peripheral blood gene sequencing of psoriasis patients found that mTOR methylation was significantly enhanced, suggesting that the mTOR gene is closely related to the pathogenesis of psoriasis (37). Buerger C et al. also reported that mTOR and the downstream signaling molecules were overexpressed in the skin lesions of psoriasis patients (38). In addition, Bürger et al. revealed that blockage of mTOR signaling with rapamycin could alleviate the symptoms of imiquimod (IMQ)-induced psoriasis in mice (39). These results imply that targeted blockage of PI3K/AKT/mTOR signaling pathway could be used as an effective strategy for the treatment of psoriasis patients.

### JAK/STAT signaling pathway

**Figure 3:**
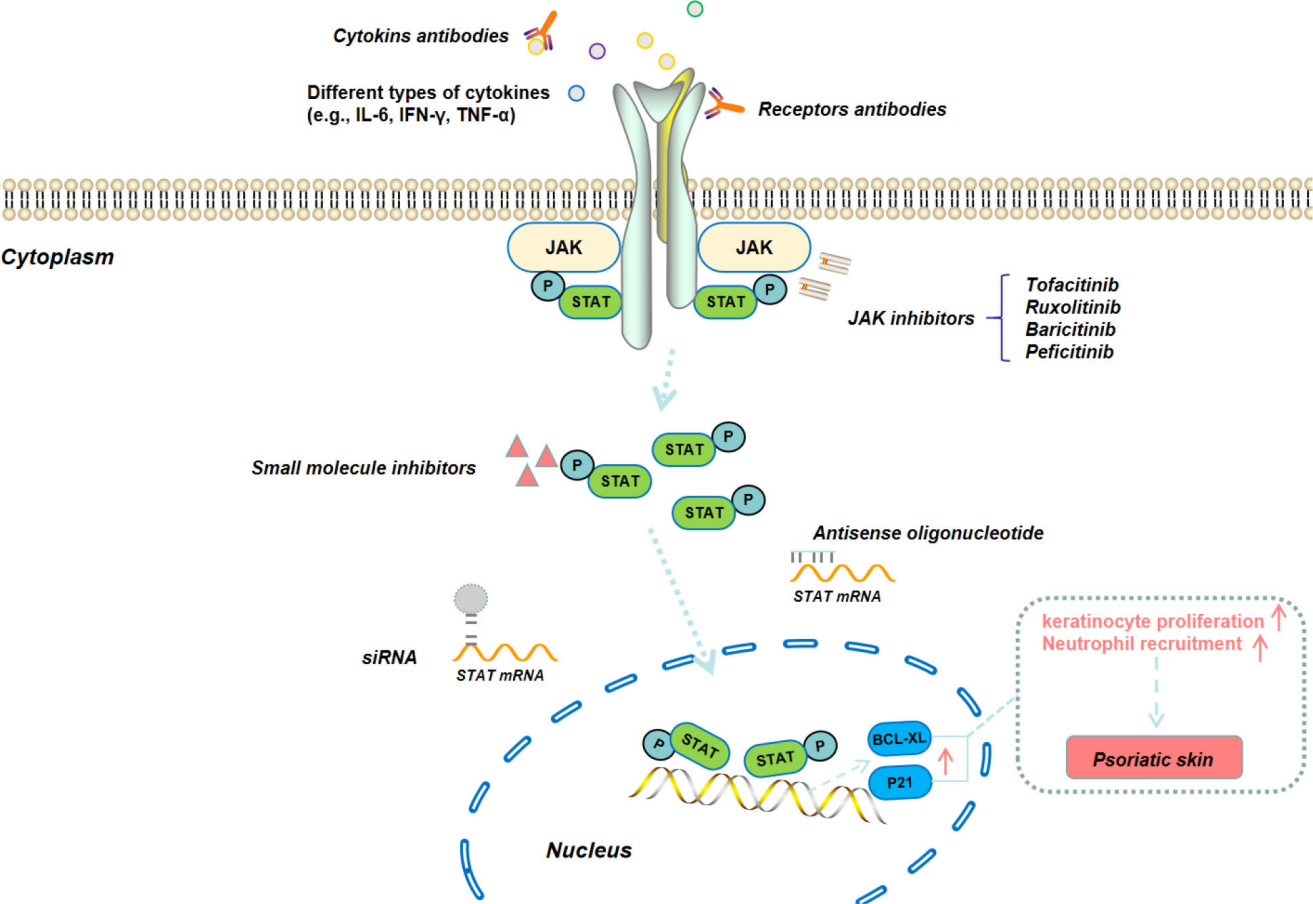
Schematic illustration of JAK/STAT signaling pathway and the corresponding therapeutic modalities for the blockage of this signaling pathway.

Janus Kinase Signal Transducer and Activator of Transcription (JAK/STAT) is an intracellular signaling pathway that enables extracellular signals to be transmitted to the nucleus through relatively few intermediate links, thereby enabling rapid cellular responses to various signals reaction (**Figure 3**) (40). The structure of the JAK/STAT signaling pathway is relatively simple, consisting of three components: tyrosine kinase-related receptors, JAK and STAT. The JAK family includes four members, *i.e.*, JAK1, JAK2, JAK3, and tyrosine kinase 2 (TYK2). The STAT family includes seven subtypes (STAT1, STAT2, STAT3, STAT4, STAT5a, STAT5b, and STAT6) (41). Many different pro-inflammatory signaling pathways converge on this pathway. Several types of inflammatory skin diseases (such as psoriasis) are closely related to the activation of JAK/STAT signaling pathway, as the activation of this pathway can enhance the secretion of various pro-inflammatory cytokines (*e.g.*, IL-17, IL-22, IL-23, and TNF-α) (42). Therefore, blocking JAK/STAT signaling pathway can impair the inflammatory response of keratinocytes and thereby alleviate the epidermal hyperplasia (43). In fact, JAK inhibitors such as tofacitinib and baricitinib have been clinically used for the treatment of psoriasis patients (44).

### MAPK/NF-κB signaling pathway

**Figure 4:**
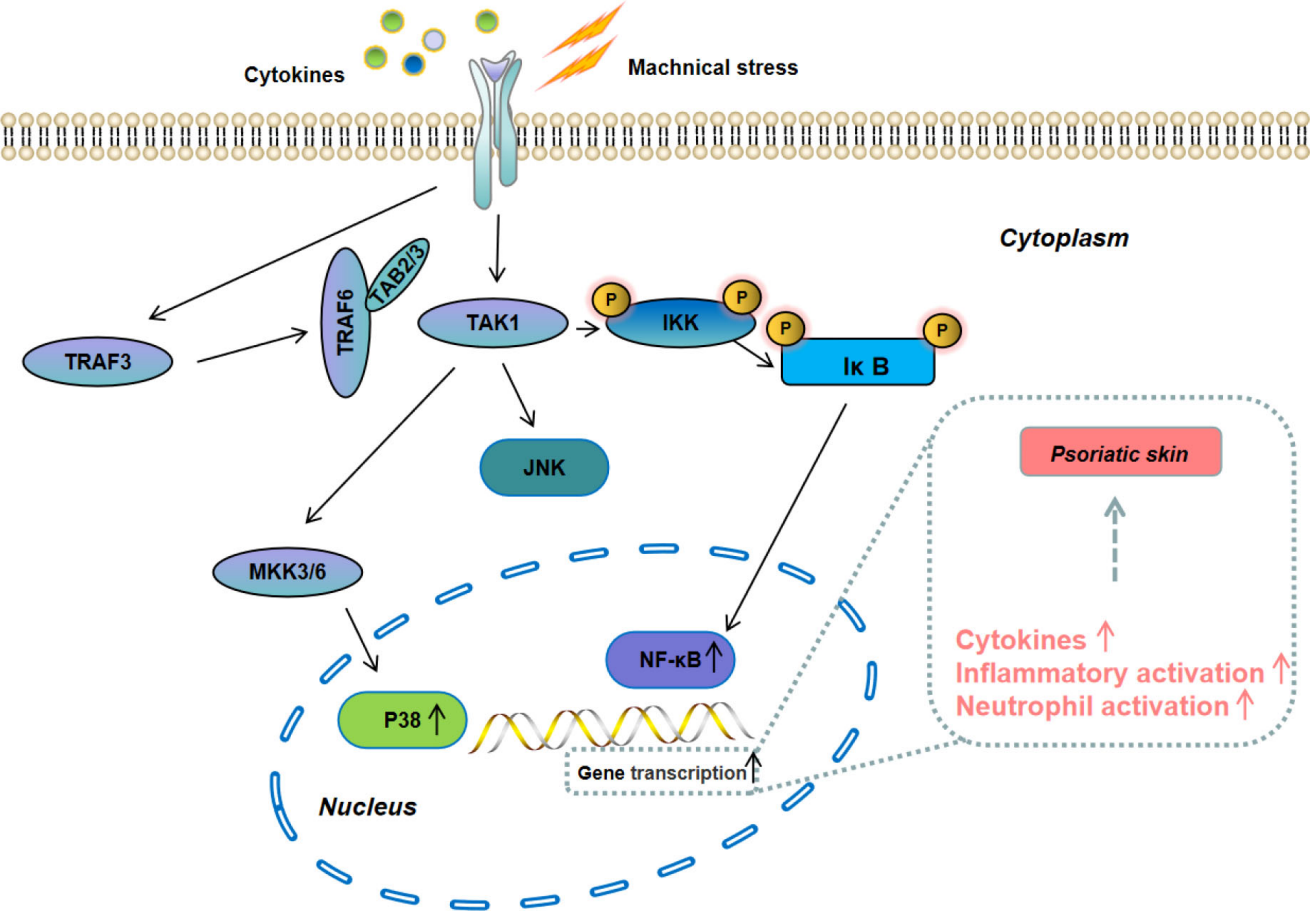
Schematic illustration of the MAPK/NF-κB signaling pathway.

Mitogen-activated protein kinase (MAPK) is a member of the serine-threonine kinases kinase family. Its signal is transmitted from the cell membrane to the nucleus and is one of the most important signaling pathways that control cells proliferation (**Figure 4**). The MAPK family includes extracellular regulated protein kinase (ERK), c-Jun N-terminal kinase (JNK), and p38 (45). Among them, the p38 MAPK signaling pathway is the most closely related to the development of progression of psoriasis, which can promote the secretion of inflammatory cytokines (*e.g*, IL-6, IL-1β, and TNF-α) to accelerate the progression of psoriasis (46, 47). NF-kB is one of the downstreams of p38 MAPK signaling pathway. Pu et al. reported that NF-κB was over-activated in psoriasis patients and blocking its activation could suppress psoriasis progression and reduce the secretion of inflammatory cytokines (48). At present, the inhibitors for MAPK/NF-κB signaling pathway have shown a great potential for psoriasis treatment and several inhibitors have been tested in experimental models and clinical trials.

### Other signaling pathways associated with psoriasis

Besides the signaling pathways described above, some other canonical signaling pathways have been also reported to involve the pathogenesis of psoriasis. For example, as a highly conserved signal transduction pathway that coordinates the regulation of epidermal cell proliferation, differentiation, and migration, dysregulation of Notch signaling pathway is closely associated with various human skin diseases including psoriasis (49–51). In addition, the activation of Wnt/β-catenin pathway could enhance the secretion of pro-inflammatory cytokines and promote the pathogenesis of psoriasis (52, 53). Moreover, peroxisome proliferator-activated receptors (PPARs), especially PPAR-γ, have been demonstrated with the ability to promote the development of psoriatic lesions and triggers IL17-related signaling cascades (54).

With the deepening of psoriasis-related pathogenesis research, some potential psoriasis-related signaling pathways have also been discovered. For example, Müller A et al. demonstrated that cyclin-dependent kinase 4 (CDK4) and CDK6 can induce the activation of STAT3 after phosphorylation of methyltransferase (EZH2) in keratinocytes, thereby inducing the production of IκBζ (a key pro-inflammatory transcription factor required for psoriatic cytokine synthesis) (55). Yan et al. recently reported that the activation of Par3/mInsc/LGN signaling pathway plays a role in the pathogenesis of psoriasis (56).

## miRNAs involved in the pathogenesis of psoriasis

MicroRNAs (miRNAs) are small and highly conserved non-coding RNA with a length of about 22 nucleotides, which can bind to specific target mRNA and play a negative regulatory role by promoting the degradation of target mRNA and inhibiting translation, and participates in biological process (*e.g.*, proliferation, differentiation, and apoptosis) (57, 58). In recent years, studies have found that a variety of miRNAs are abnormally expressed in psoriatic skin lesions and plasma, and different miRNAs and their target genes can participate in the occurrence and development of psoriasis through different signal transduction pathways (59, 60). So far, there have been some related studies to reveal the important role of miRNAs in regulating the development and progression of psoriasis.

miR-203 was the first miRNA reported in psoriasis patients (61). A study showed that miR-203 could inhibit the expression of SOCS3 by directly binding to the 3’-UTR of SOCS3 and promoted the degradation of SOCS3 mRNA, thereby activating the JAK2/STAT3 signaling pathway to promote the progression of psoriasis. MiR-155 is another miRNA that plays a critical role in various physiological and pathological processes including hematopoietic lineage differentiation, immunity, inflammation, cancer and cardiovascular disease (62). García-Rodríguez et al. found that the expression level of miR-155 in peripheral blood mononuclear cells was positively correlated with the psoriasis area and severity index (PASI) score of psoriasis patients (63). Recently, Lovendorf et al. reported that miR-223 and miR-143 were also up-regulated in psoriasis patients and positively correlated with PASI score (64). Besides the up-regulated miRNAs described above, some miRNAs have been found to be down-regulated in the tissues of psoriasis and also involve the pathogenesis of psoriasis. For example, miR-320b is down-regulated in the tissues of psoriasis and could participate in the pathogenesis of psoriasis *via* regulating the STAT3 and SAPK/JNK signaling pathways (65). In addition, MiR-205-5p has been also reported to be down-regulated in psoriatic skin tissue and could negatively regulate the Wnt/β-catenin signaling pathway to alleviate IMQ-induced psoriasis symptoms (66).

To date, a large number of miRNAs have been reported to play crucial regulatory roles in the pathogenesis of psoriasis *via* multiple molecular mechanisms. However, the regulatory mechanisms of some miRNAs are still unclear, and much more effects need to be paid to elucidate the inherent reasons.

## NPS-mediated drug delivery for psoriasis therapy

At present, topical and systemic treatments are still the main clinical treatment modalities for psoriasis. In general, patients with mild-to-moderate psoriasis usually receive topical therapy, while systemic therapy is applied for the treatment of patients with moderate-to-severe psoriasis by using small molecule drugs and biologics (67). The commonly used small molecule drugs include cyclosporine A, methotrexate, sulfasalazine, leflunomide, apremilast, and retinoids (8, 9), while the biologics mainly include monoclonal antibodies that can target the cytokines-related signaling pathways, especially TNF-α signaling pathway and IL-23/Th17 axis. Up to now, more than ten types of biologics have been approved for psoriasis therapy. Among them, four types biologics (*i.e.*, adalimumab, infliximab, certolizumab, and etanercept) are used to target TNF-α signaling pathway (68). In addition, three anti-IL17 biologics (ixekizumab, secukinumab, and brodalumab) and four anti-IL23 biologics (ustekinumab, guselkumab, risankizumab, and tildrakizumab) have been also marketed for the treatment of psoriasis *via* blocking the IL-23/Th17 axis. Currently, although small molecule drugs and biologics have achieved great success in the treatment of psoriasis, some key issues remain to be resolved, especially the toxic and side effects.

In the past two decades, the application of NPs for drug delivery has introduced exciting opportunities to improve the therapeutic outcomes of various diseases including psoriasis, showing the advantages of improving the drug stability, improving the drug accumulation in the disease sites, and reducing the toxic and side effects. Inspired by these unique advantages, numerous NPs have been designed and developed for the delivery of various types of therapeutics drugs (*e.g.*, small molecules and nucleic acid drugs) and psoriasis therapy.

### NPs-mediated delivery of small molecule drugs for the treatment of psoriasis

**Figure 5:**
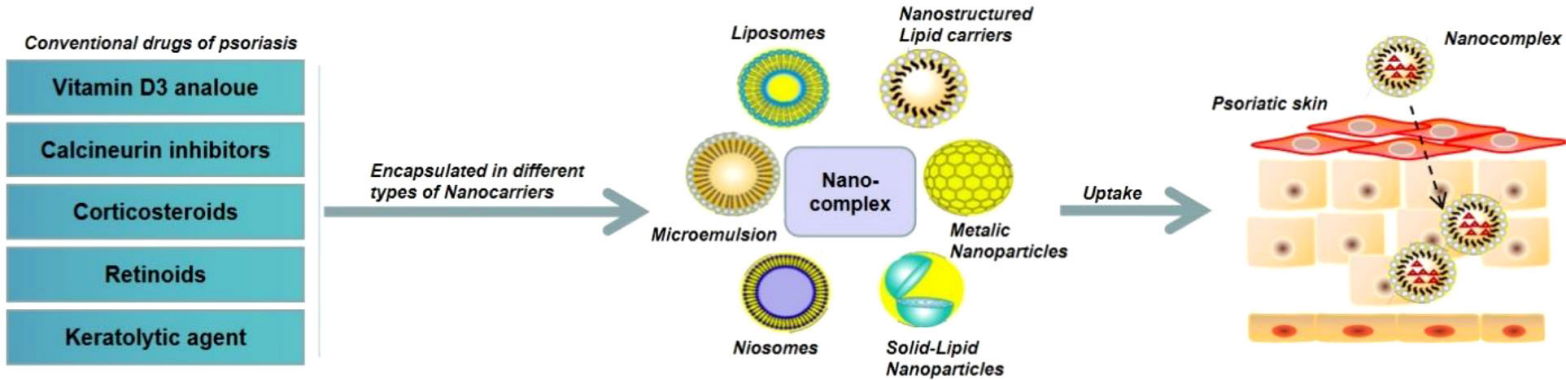
Schematic illustration of NPs-mediated delivery of small molecule drugs for the treatment of psoriasis.

Corticosteroids, a type of lipophilic drugs that show anti-inflammatory, anti-proliferative, and immunosuppressive effects, have been widely used for the clinical treatment of various skin diseases such as psoriasis, dermatitis, and eczema (69). However, corticosteroids could not penetrate into diseased skins and patients are usually resistant to corticosteroids treatment. Recent studies have shown that encapsulation of corticosteroids into NPs could significantly improve their bioavailability (70). Cyclosporine A (CsA), a calcineurin inhibitor, has shown good therapeutic effect in the treatment of psoriasis. In order to reduce the toxicity of CsA, Lapteva et al. encapsulated CsA into polymeric NPs made with the methoxy-poly(ethylene glycol) di-(hexyl-substituted polylactide) (mPEG-dihexPLA) polymer, which could not only increase the water solubility of CsA by around 518-fold, but also significantly enhance the therapeutic effect of CsA with reduced toxic and side effects (71). Recently, Walunj et al. reported a liposome gel containing CsA-loaded cationic liposomes, which could dramatically reduce psoriasis symptoms and the level of psoriatic cytokines (*e.g.*, TNF-α, IL-17, and IL-22) after topical administration to an IMQ-induced psoriatic plaque model (72). Methotrexate (MTX) is the first-line anti-proliferative drug for the treatment of moderate to severe psoriasis, but long-term administration of MTX can induce severe side effects. Avasatthi et al. developed a nanogel consisting of a nanostructured lipid carrier loaded with MTX (MTX-NLC) that could prolong MTX release and significant reduce PASI scores in the IMQ-induced mice model (73). Özcan et al. coupled MTX with gold NPs (MTX-GNP) and demonstrated that MTX-GNP could accomplish better anti-inflammatory effect and tolerability than MTX alone *via* reducing the infiltration of CD4^+^ T cells, γδ T cells, and neutrophils in the skin tissues of psoriasis (74). Besides corticosteroids, CsA, and MTX, many other small molecule drugs have been also loaded into various types of NPs for the treatment of psoriasis (**Figure 5**).

### NPs-mediated delivery of nucleic acid drugs for the treatment of psoriasis

**Figure 6:**
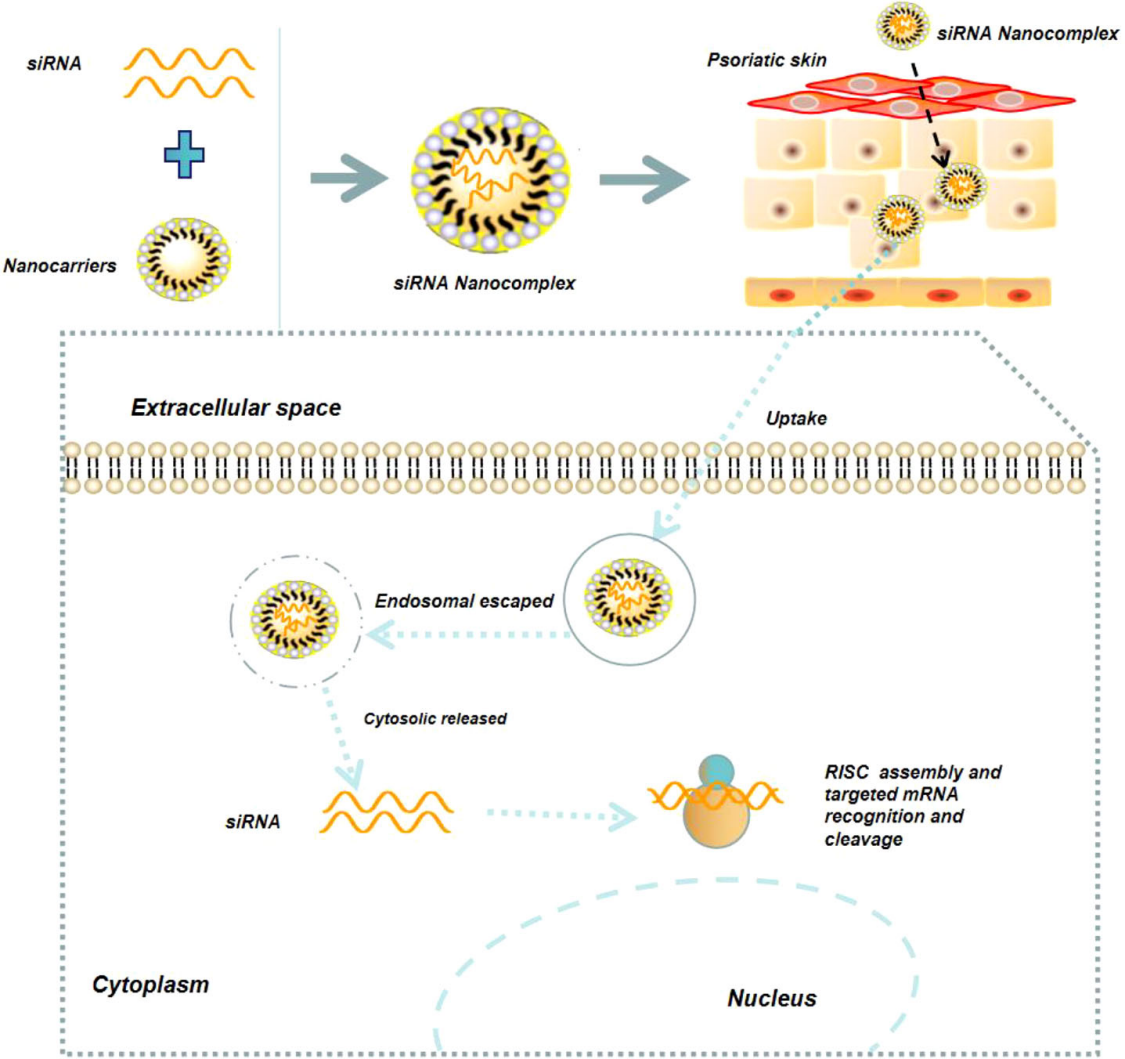
Schematic illustration of NPs-mediated delivery of nucleic acid drugs (*e.g.*, siRNA) for the treatment of psoriasis.

Nucleic acid drugs mainly include miRNA, siRNA, mRNA, and DNA. In recent years, nucleic acid drugs have been widely used for psoriasis therapy, due to their ability to inhibit the expression of disease-promoting target genes or up-regulate the disease-inhibiting target genes (59, 60, 75). For example, because miR-31 is highly over-expressed in psoriatic skin and could enhance NF-κB signaling pathway and IL-1β production, specific inhibition of miR-31 by its antisense strand could dramatically suppress the progression of psoriasis (60, 76). However, because nucleic acid drugs are negatively charged biomacromolecules, specific delivery tools are required to improve their cytosolic delivery (**Figure 6**) (77). Feng et al. developed a biomimetic recombinant high-density lipoprotein (rHDL) nanogel for the delivery of miR-210 antisense (NG-anti-miR-210) (78). Local administration of NG-anti-miR-210 could significantly down-regulate miR-210 expression, reduce the mRNA level of IL-17A and INF-γ, and alleviate the skin lesion symptoms of psoriasis. Marepally et al. used cationic lipid NPs to concurrently encapsulate STAT3 siRNA (siSTAT3) and TNF-α siRNA (siTNF-α) and demonstrated that these NPs could synergistically alleviate the skin lesion symptoms of psoriasis *via* inhibiting the expression of STAT3, TNF-α, and IL-23 (79). Recently, Boakye et al. also employed lipid NPs to co-encapsulate erlotinib and IL36α siRNA, and revealed that this delivery system significantly reduce the secretion of inflammatory factors (IL36α, IL23, IL17, and TNF-α) in psoriatic plaques, thereby alleviating the symptoms of psoriasis (80).

**Figure 7:**
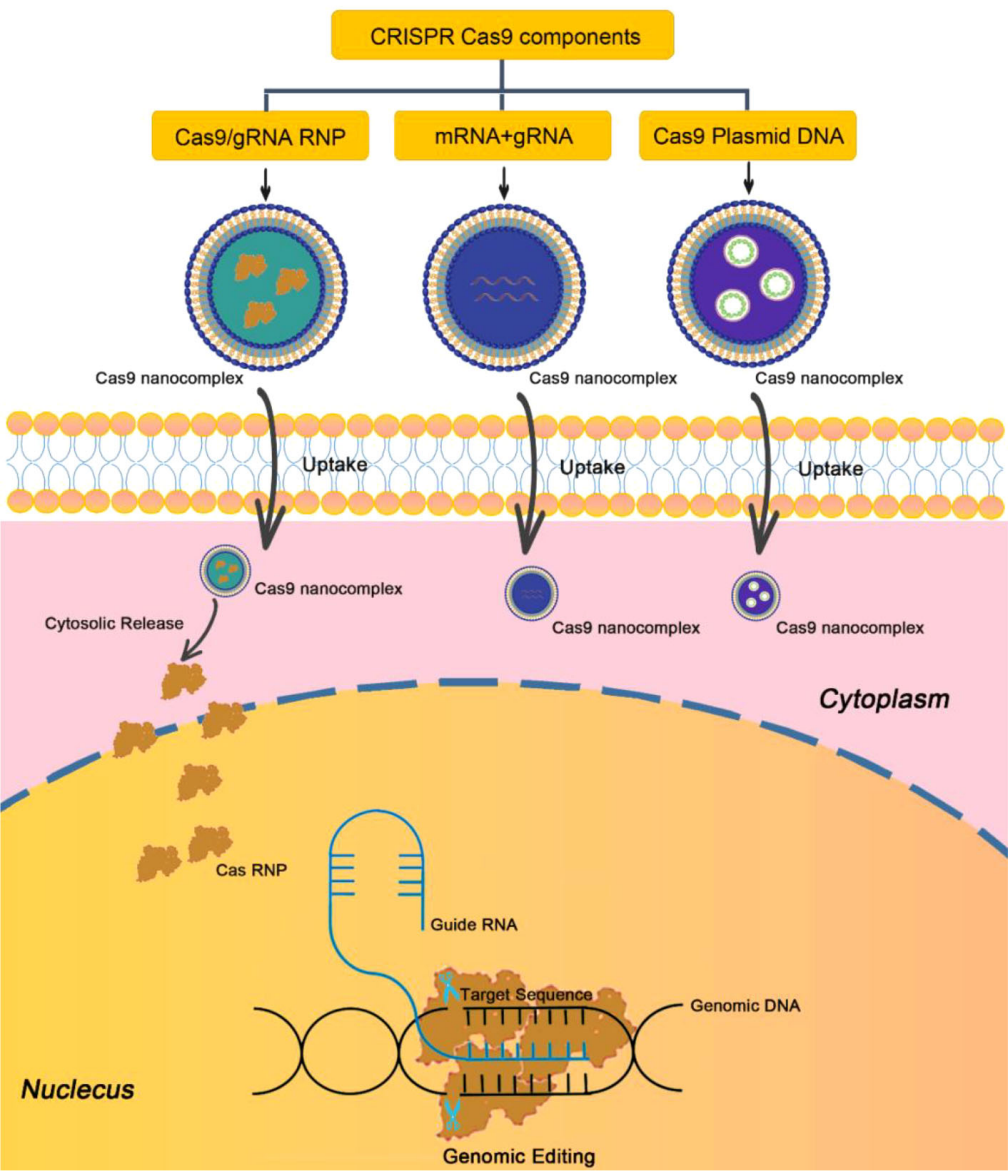
Schematic illustration of CRISPR-Cas9 technique for gene editing.

In recent years, gene editing based on clustered regularly interspaced palindromic repeats (CRISPR) is one of the great technologies discovered in the biological field. Currently, CRISPR-Cas9 technique has become one of the most powerful tools for disease treatment (81). The mechanism of CRISPR-Cas9 technique is to recognize the target gene sequence through artificially designed small guide RNA (sgRNA) and then guide the Cas9 protease to cut the DNA double-strand for gene silencing (**Figure 7**). In recent years, CRISPR-Cas9 technique has been used for the treatment of psoriasis. For example, Swindell et al. employed CRISPR-Cas9 technique to knock out Myd88 gene in epidermal keratinocytes, and indicated that Myd88 knockout could significantly reduce the expression of its downstream IL-1β and IL-36, which are two important factors regulating the inflammatory response of psoriasis (82). Recently, Wan et al. developed a dissolvable microneedle (MN) patch loading with Cas9 ribonucleoprotein (RNP) targeting pyrin domain-containing 3 (NLRP3) and dexamethasone (Dex)-containing polymeric NPs for the treatment of psoriasis (83). NLRP3 is an inflammasome implicated in a variety of inflammatory and autoimmune skin diseases including psoriasis and atopic dermatitis. Therefore, co-delivery of Cas9-NLRP3 and Dex could significantly alleviate the symptoms of psoriasis and improve overall inflammatory activity compared to the single treatment with Cas9-NLRP3 or Dex (83).

### NPs with anti-inflammatory effect for the treatment of psoriasis

It has been demonstrated that the pro-inflammatory cytokines play important roles in the pathogenesis of psoriasis. Therefore, design and development of NPs with anti-inflammatory effect has been recognized as a promising strategy for the treatment of psoriasis. Up to now, various metal NPs especially silver nanoparticles (Ag NPs) and gold nanoparticles (Au NPs) have been developed for the treatment of psoriasis with powerful anti-inflammatory effect (84–86). For example, Sumbayev et al. firstly reported Au NPs could weaken IL-β-induced pro-inflammatory response, which thus provides the possibility to use Au NPs for psoriasis treatment (87). Han et al. recently developed a type of modified Au NPs (Au@PEG-octadecyI_30%_ NPs) with an average size of less than 15 nm, which could penetrate the stratum corneum and preferentially enter keratinocytes when applied to a mouse model of IMQ-induced psoriasis. More importantly, these modified Au NPs could reduce the pro-inflammatory response induced by the IL-17 signaling pathway and show the similar therapeutic efficacy as standard steroids and vitamin D analogs without causing hair loss and skin wrinkles (86). Recently, Crisan et al. prepared the nanocomplexes polyphenols-rich natural extracts (Cornus mas, CM) and Ag NPs (Ag-NPs-CM) or AuNPs (Au-NPs-CM), and demonstrated that these NPs can reduce the secretion of pro-inflammatory factors (*e.g.*, IL-12 and TNF-α) in psoriasis by blocking of the NF-κB signaling pathway in macrophages (88). Besides the metal NPs, Chen et al. recently reported that polymeric NPs composed of alantolactone-modified chitosan and hyaluronic acid (CHALT) could effectively inhibit the proliferation of keratinocytes *via* ROS-mediated loss of mitochondrial membrane potential and apoptosis. In addition, CHALT could also weaken the inflammatory response triggered by IL-6 and JAK/STAT3 signaling pathway in keratinocytes, thereby inhibiting the progression of psoriasis (89).

### Microneedles-mediated drug delivery for the treatment of psoriasis

Transdermal drug delivery is an effective route for the treatment of skin diseases, because it allows direct targeting of lesions on the skin and reduces the side effects associated with systemic drug delivery (90–93). However, the skin barrier-stratum corneum could greatly impair the bioavailability of transdermal drug delivery. Microneedles is a type of new drug delivery systems developed in recent years, which can significantly improve the permeability and increase the bioavailability of therapeutic agents by piercing the stratum corneum and creating a large number of reversible microchannels in a minimally invasive manner. Due to these unique advantages, microneedles have been widely used as effective transdermal drug delivery tools for the treatment of skin diseases including psoriasis (90, 91, 94). Du et al. developed a microneedle patch made with hyaluronic acid (HA) and encapsulated MTX into the patch, which could effectively penetrate the IMQ-induced mouse epidermis and impair psoriasis-like skin inflammation in mice (95). Recently, Men et al. encapsulated tacrolimus (TAC) into a sodium hyaluronate-based microneedle patch, and demonstrated that this drug delivery system could significantly enhance the retention of TAC in the skin tissues and alleviate the symptoms of psoriatic skin *via* impairing the secretion of inflammatory cytokines such as TNF-α and IL-23 (96).

## Conclusion and outlook

Although the pathogenesis of psoriasis is diverse and complex, cytokine inflammatory loops, cell signaling pathways, and epigenetic factors such as miRNAs have been widely recognized as the important factors regulating the development and progression of psoriasis. Based on these regulatory mechanisms, a variety of therapeutic drugs including small molecule drugs and biologics have been designed and approved for clinical treatment of psoriasis. Although these drugs have achieved great success in clinic, much more efforts need to be paid to address their drawbacks, especially the toxic and side effects. The use of NPs for drug delivery has provided the valuable chances to promote the therapeutic outcomes of various types of drugs and thus provided innovative insights into the treatment of psoriasis. However, the clinical translation of these nanodrugs is still challenged and more efforts are required to promote their clinical use, especially needing to solve the difficulty in controllable and reproducible synthesis of nanodrugs and their scalable manufacturing. With rapid development of molecular biology and nanomedicine, we believe that more types of therapeutics will be definitely developed and marked for the treatment of psoriasis.

## Author contributions

QS, RLiu, ST, and JF wrote the manuscript. QS, XX, JF, and RLi revised the manuscript. All authors contributed to the article and approved the submitted version.
